# Performances of Transcritical Power Cycles with CO_2_-Based Mixtures for the Waste Heat Recovery of ICE

**DOI:** 10.3390/e23111551

**Published:** 2021-11-21

**Authors:** Jinghang Liu, Aofang Yu, Xinxing Lin, Wen Su, Shaoduan Ou

**Affiliations:** 1 School of Energy Science and Engineering, Central South University, Changsha 410083, China; ljhupup@csu.edu.cn (J.L.); yuaf923@csu.edu.cn (A.Y.); shaoduan@csu.edu.cn (S.O.); 2 CTG (China Three Gorges Corporation) Science and Technology Research Institute, Beijing 100038, China; lin_xinxing@ctg.com.cn

**Keywords:** CO_2_-based mixtures, transcritical power cycles, waste heat recovery, thermodynamic analysis

## Abstract

In the waste heat recovery of the internal combustion engine (ICE), the transcritical CO_2_ power cycle still faces the high operation pressure and difficulty in condensation. To overcome these challenges, CO_2_ is mixed with organic fluids to form zeotropic mixtures. Thus, in this work, five organic fluids, namely R290, R600a, R600, R601a, and R601, are mixed with CO_2_. Mixture performance in the waste heat recovery of ICE is evaluated, based on two transcritical power cycles, namely the recuperative cycle and split cycle. The results show that the split cycle always has better performance than the recuperative cycle. Under design conditions, CO_2_/R290(0.3/0.7) has the best performance in the split cycle. The corresponding net work and cycle efficiency are respectively 21.05 kW and 20.44%. Furthermore, effects of key parameters such as turbine inlet temperature, turbine inlet pressure, and split ratio on the cycle performance are studied. With the increase of turbine inlet temperature, the net works of the recuperative cycle and split cycle firstly increase and then decrease. There exist peak values of net work in both cycles. Meanwhile, the net work of the split cycle firstly increases and then decreases with the increase of the split ratio. Thereafter, with the target of maximizing net work, these key parameters are optimized at different mass fractions of CO_2_. The optimization results show that CO_2_/R600 obtains the highest net work of 27.43 kW at the CO_2_ mass fraction 0.9 in the split cycle.

## 1. Introduction

### 1.1. Background

Recently, with the rapid development of economy, the demand for energy in various industries has exploded. Although there exist many types of energy sources, such as fossil energy, solar energy, and geothermal energy, fossil energy is still dominant. So far, CO_2_ produced by fossil fuel combustion has caused serious global warming, which attracts the attention of all countries in the world. Thus, it is urgent to improve the utilization efficiency of fossil fuel. As one of the major application fields of fossil fuel combustion, internal combustion engines (ICEs) consume a large amount of fossil oil every year. However, only 30–40% of the energy is converted into useful work [1], and a large amount of fuel heat is released through the exhaust gas and coolant of the vehicle. In general, the waste gas temperature of ICE can be up to 400–700 °C and the temperature of engine coolant is about 80–90 °C. Therefore, the recovery of high temperature exhaust heat can greatly improve the ICE efficiency and reduce fuel consumption. So far, various thermodynamic cycles have been proposed for efficiently recovering waste heat from ICE. Among these proposed cycles, the most representative technology is the organic Rankine cycle (ORC) [2,3,4], which has the advantages of simple structure and easy maintenance.

### 1.2. ORC

So far, there are many published pieces of literature to investigate the ORC application in ICE experimentally and theoretically. For example, with the aim to improve the ICE efficiency, Zhao et al. [5] applied ORC to recover the exhaust heat. The theoretical results indicated that the ICE output power increases by 4.13 kW. Uusitalo et al. [6] examined experimentally the influences of small-scale high-temperature ORC on the performance under different operating conditions. The experimental results showed that the maximum cycle power output is 6 kW with the turbine operating in the rotational speed range of 12,000 rpm–31,000 rpm. Furthermore, Tian et al. [7] proposed a novel ORC system and analyzed 20 working fluids’ thermo-economic performance. The results indicated that R141b, R123, and R245fa not only have the largest cycle efficiency and net work, but also possess the lowest electricity production cost. In addition to the commonly used subcritical ORC, the transcritical ORC is also considered in the ICE waste heat recovery. Being different from the subcritical ORC, the transcritical ORC makes the working fluid absorb heat at a supercritical state. For instance, Mohammadkhani et al. [8] applied a transcritical two-loop ORC to utilize the waste heat from ICE. Toluene and R143a were considered as working fluids. The simulation results showed that the system net power is 24.93 kW, and the specific investment cost is 4361$/kW. Based on the existing published literatures, Wang et al. [9] comprehensively reviewed the technology to recover the waste heat from ICE, and provided different methods of system design to improve efficiency. From the simulation results, they found that the heat recovery system can increase powertrain efficiency by 30% under specific operating conditions. However, although many researches on ORC have been conducted to recover the ICE waste heat in the last decade, the employed organic fluids are usually flammable, toxic, or of high global warming potential (GWP). In addition, at high temperatures, organic fluids are easily decomposed, thus greatly limiting the ORC efficiency improvement in the waste heat recovery of ICE.

### 1.3. Transcritical Power Cycle with CO_2_

To address the above issues, many scholars have used CO_2_ to replace the organic fluid in the field of waste heat recovery. Compared with the organic fluid, CO_2_ has low GWP and exhibits high stability, with being non-flammable and non-toxic. Furthermore, because of the low critical temperature (31.1 °C), CO_2_ is preferred to be applied in the transcritical Rankine cycle to recover the ICE waste heat. Compared to the ORC, the CO_2_ power cycle has obvious advantages: (1) the high density of supercritical CO_2_ state can greatly improve the compactness of heat exchangers, thus resulting in a smaller system footprint [10]; (2) the CO_2_ power cycle can be started up quickly, and has a fast response speed [11]. This cycle is more suitable to recover the ICE waste heat. In view of these advantages, extensive research has yet been carried out to investigate the CO_2_ power cycle performance in the waste heat recovery. For instance, Shi et al. [12] established an experimental system of the transcritical CO_2_ power cycle to recover the diesel engines waste heat. The effects of the pressure ratio, engine speed, and pump speed on system performance were experimentally conducted. The experimental results indicated that the net work (2.05 kW) can be expected to be obtained with 1300 rpm of engine and 80 rpm of pump, and the expected thermal efficiency is 4.3% with 1300 rpm of engine and 70 rpm of pump. Li et al. [13] investigated a transcritical CO_2_ power cycle with a preheater and recuperator to recover the engine waste heat, and analyzed in detail the temperature disturbance during the heating process. The results illustrated that the reduction of temperature interference can increase the cycle thermal efficiency. Meanwhile, the optimized net work increases from 14.7 kW to 19.0 kW. In addition, a transcritical CO_2_ cycle test to recover low-grade waste heat was developed by Li et al. [14]. The effect of working fluid mass flow on the system performance was investigated. It was found that with the increase of working fluid mass flow, the overall system pressure increases and output power decreases. In terms of the performance comparison between CO_2_ and organic fluid, Baik et al. [15] optimized the net works of transcritical CO_2_ and R125 cycles to recover the low-grade heat. The results showed that R125 performs better, with 14% higher power than CO_2_. Moreover, the advantage of fast response of the transcritical CO_2_ power cycle has been studied in relevant literature [16]. It was found that the response speed of a simple CO_2_ cycle is four times faster than that of R123 cycle.

### 1.4. Transcritical Power Cycle with CO_2_-Based Mixture

For the engineering application of the transcritical CO_2_ power cycle, there still exist many challenges. For example, due to the fact that the CO_2_ critical temperature approaches the ambient temperature, it is very difficult to effectively condense CO_2_ by cooling water or air, especially in the waste heat recovery of ICE. In addition, the CO_2_ cycle pressure is relatively high, thus resulting into a high manufacturing cost and a great safety risk during the practical application. To solve the above issues effectively, CO_2_ is proposed to mix with the organic fluid, thus forming the CO_2_-based mixture [17]. In comparison with the pure CO_2_, the CO_2_-based mixture can increase the critical temperature to extend the condensation temperature scope of the transcritical system. Meanwhile, at the same bubble temperature, the CO_2_-based mixture has a lower condensation pressure than CO_2_. Furthermore, the mixture has advantages over the organic fluids in the aspect of safety. The relevant study [18] has shown that the added CO_2_ can effectively reduce the organic fluid flammability. By conducting experiments on the flammability limit, it was found that when the CO_2_ molar fraction is larger than 0.3, CO_2_/R290 is non-flammable [19]. Therefore, the researches on the transcritical cycle performance of CO_2_-based mixtures have aroused the interest of many scholars. For instance, Dai et al. [20] applied a transcritical cycle with CO_2_-based mixtures to convert low-grade heat. It was found that the cycle thermal efficiency is maximized when the carbon dioxide mass fraction is about 0.5. Aiming at the geothermal water with 100–150°C, Wu et al. [21] applied CO_2_-based mixtures into a transcritical cycle to produce power. Six refrigerants (R152, R161, R290, R1234yf, R1234ze, R1270) were selected to be mixed with CO_2_. The results revealed that the best thermo-economic performance of CO_2_/R161(0.3/0.7) is achieved when the CO_2_ molar fraction is 0.3. When the condensation temperature is 10–20 °C, the net work of CO_2_/R161(0.3/0.7) is increased by 14.43–50.46% over that of pure CO_2_. For the utilization of low-grade heat, Chen et al. [22] implemented a thermodynamic analysis on the transcritical cycle using CO_2_/R32 as a working fluid. The analysis results showed that CO_2_/R32 can a achieve high thermal efficiency of 12.6–18.7%. In terms of ICE waste heat recovery, Shu et al. [23] conducted the transcritical power cycle on the waste heat recovery of ICE, based on the CO_2_ mixture. In this cycle, a preheater and a recuperator were employed. The corresponding cycle performance of various mixtures were investigated. The results showed that CO_2_/R32(0.3/0.7) has the largest output work among the considered mixtures, when the condensation temperature is below 40 °C. As for the heat transfer area, the cycle with CO_2_/R32(0.3/0.7) has a lower value than that of CO_2_ cycle. Meanwhile, Shu et al. [24] also studied the transcritical cycle dynamic performance with CO_2_-based mixtures to recover waste heat from the truck engine. It was found that as the CO_2_ fraction increases, the system responds faster. On this basis, many scholars further studied the control strategy for the transcritical power cycle with CO_2_-based mixtures. Aiming at the waste heat recovery of heavy-duty diesel engines, Wang et al. [25] adopted three control strategies (constant temperature, constant pressure, and optimal control strategies) to reach system stability. From the simulated results, it was found that under varying conditions of ICE, the system stability with optimal control or constant pressure control is better than that with constant temperature control. With the optimal control strategy, the system can output the highest net work.

### 1.5. Purpose of This Work

From the existing literatures, it can be found that only a few studies have compared the performance of different CO_2_-based mixtures in cycles with relatively simple structures. However, with the development of advanced cycles, for the utilization of CO_2_-based mixtures in the ICE waste heat recovery, additional studies are needed to further compare the various CO_2_-based mixtures’ performance in advanced cycles. Furthermore, aiming at the condensation of the transcritical cycle to recover the ICE waste heat, most researchers [24,26] just fix the condensation temperature at 20–35 °C to conduct a performance analysis. However, considering the ambient temperature, this temperature range is difficult to achieve in the mobile engine. In fact, the condensation temperature is strongly dependent on the used condensation method [27]. In total, there are three methods to condense the fluid in the waste heat recovery of ICE. (1) A separate low-temperature cooling package is employed to achieve condensation. In this case, the condensation temperature of working fluid can be low to 30 °C. (2) The working fluid is condensed by the intermediate medium. The corresponding condensation temperature can range from 50 °C to 70 °C. (3) The engine coolant is employed as the heat sink of cycle. Thus, the condensation temperature can be up to 90 °C. 

Aiming at the deficiencies of the existing researches, this study employs two transcritical power cycles and applies five CO_2_-based mixtures to recover the waste heat from ICE. The considered cycles include the recuperative cycle and the split cycle. Compared with the recuperative cycle, the split cycle can deeply recover the high-temperature waste heat. As for the CO_2_ mixtures, five organic fluids, namely R290, R600a, R600, R601a, and R601, are selected to mix with CO_2_. In addition to the performance comparison of these mixtures in the two cycles, the effects of turbine inlet temperature (*T*_3_), turbine inlet pressure (*P*_h_), condensation temperature (*T*_1_), and mass fraction of CO_2_ on system performance are discussed. On this basis, the split ratio (*SR*), *T*_3_, and *P*_h_ are simultaneously optimized using the genetic algorithm (GA) with the objective of maximizing net work (*W*_net_). 

## 2. Cycle Layout

**Figure 1:**
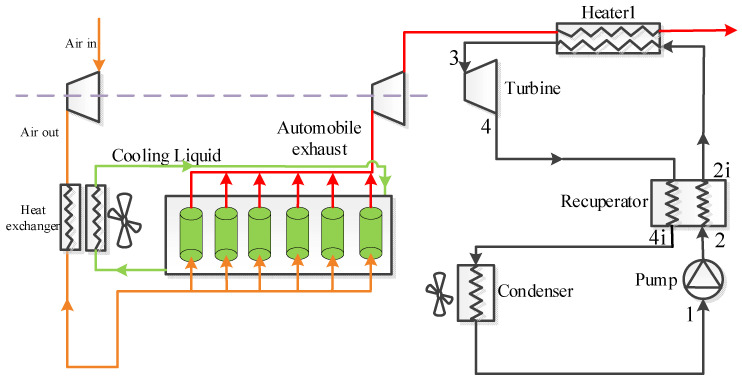
Systematic diagram of the recuperative cycle.

**Figure 2:**
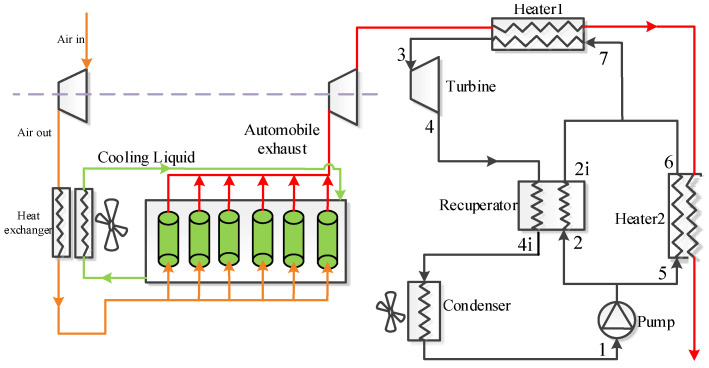
Systematic diagram of the split cycle.

**Figure 3:**
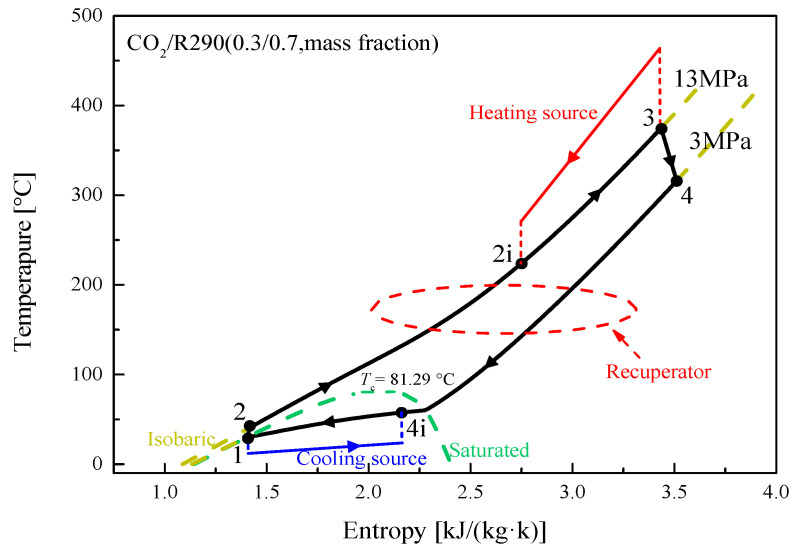
T-s diagram of the recuperative cycle under design conditions.

In this study, two transcritical cycles, namely the recuperative cycle and split cycle, are considered. The corresponding layouts are respectively shown in Figure 1 and Figure 2. It can be observed that besides the four basic components (working fluid pump, heater, turbine, and condenser), a recuperator is added at the pump outlet to recover the energy of the outlet fluid from the turbine in the recuperative cycle. This happens so that the cycle efficiency (*η*_th_) and exergy efficiency (*η*_ex_) can be increased. For the recuperative cycle, the detailed process description can be referred to in these pieces of literature [28,29]. The corresponding T-s diagram is provided in Figure 3 under design conditions. Figure 3 shows that the exhaust gas outlet temperature is still as high as 253 °C. This is because there exists a large amount of recuperative heat, thus resulting in a high temperature of the working fluid entering the heater.

**Figure 4:**
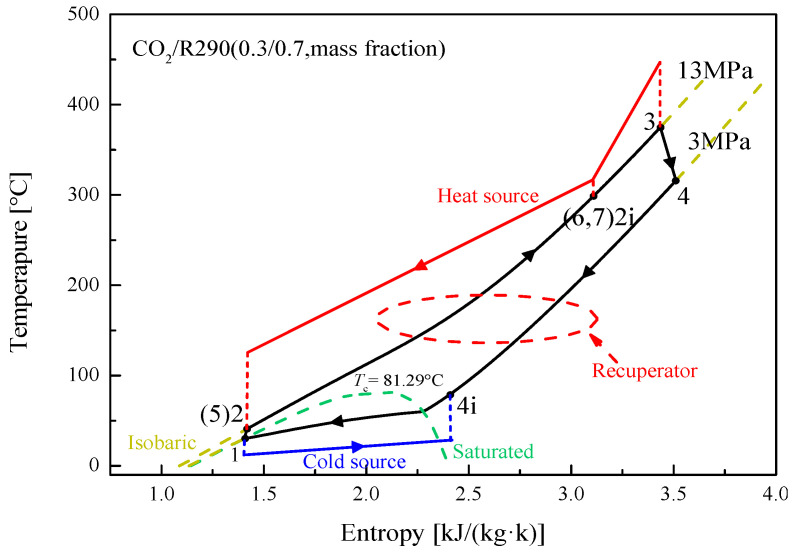
T-s diagram of the split cycle under design conditions.

As for the split cycle, Heater2 is added at the outlet of Heater1 to further extract the waste heat. After being compressed in the pump, the flow is separated into two streams. One stream enters into the recuperator to conduct a heat exchange with the high-temperature fluid at the turbine outlet. Another enters Heater2 to recover the waste heat. Thereafter, two flows are mixed at the inlet of Heater1 to continue absorbing exhaust gas heat. The remaining processes of this cycle are similar to those of the recuperative cycle. Figure 4 presents the corresponding T-s diagram of the split cycle under design conditions. The figure shows that the split cycle can make the waste gas temperature decrease to 139 °C.

For the ICE, a turbocharged technology is used, and a six-cylinder engine is considered to produce waste gas. The inhaled fresh air is firstly compressed and exchanges heat with the jacket water, and then enters the cylinder to mix with the gasoline. After combustion, the generated waste gas is exhausted. The discharged gas enters the turbine to provide work for the air compressor. As shown in Figure 1 and Figure 2, the working fluid of the power cycle is directly condensed by the air, to get the lowest condensation temperature. As for the other two condensation methods, the readers can refer to the literature [27]. 

## 3. Thermodynamic Modeling and Optimization

### 3.1. Thermodynamic Modeling

To compare the thermodynamic performance of CO_2_ mixtures in the considered two cycles, mathematical models are established. Before giving the used equations, the following assumptions are applied to simplify the modeling.

1. Without considering the variation of the system kinetic and potential energy;

2. The system operates stably and without considering the heat loss and pressure drop of pipes and components;

3. The counter-flow heat exchanger is considered in the simulation;

4. The compression and expansion processes are characterized by isentropic efficiencies.

According to the above assumptions, energy equations of each component are listed in Table 1 for the two cycles.

The corresponding net work and cycle efficiency are calculated by:

(1) $$W_{\mathrm{net}\;}=W_{t}-W_{P}$$

(2) $$\eta_{\mathrm{th}}=\left\{ \begin{array}{l} \begin{array}{cc} \frac{W_{\mathrm{net}\;}}{Q_{\mathrm{Heater}1}} & \;\;\;\;\;\;\;\;\;\;\;\;\;\;\;\mathrm{Recuperative}\;\mathrm{cycle} \end{array}\\ \begin{array}{cc} \frac{W_{\mathrm{net}\;}}{Q_{\mathrm{Heater}1}+Q_{\mathrm{Heater}2}} & \;\;\;\;\mathrm{Split}\;\mathrm{cycle} \end{array} \end{array}\right.$$

In addition to the cycle efficiency, the waste heat recovery efficiency (*η*_r_) can be expressed as below:

(3) $$\eta_{r}=\left\{ \begin{array}{l} \begin{array}{cc} \frac{Q_{\mathrm{Heater}1}}{m_{g}\left(h_{g,\mathrm{in}}-h_{g}(T_{0})\right)} & \;\mathrm{Recuperative}\;\mathrm{cycle} \end{array}\\ \begin{array}{cc} \frac{Q_{\mathrm{Heater}1}+Q_{\mathrm{Heater}2}}{m_{g}\left(h_{g,\mathrm{in}}-h_{g}(T_{0})\right)} & \;\mathrm{Split}\;\mathrm{cycle} \end{array} \end{array}\right.$$

Before establishing the exergy equations, the specific exergy value at each steady point (i) is calculated as [30,31]:

(4) $$e_{i}=\left(h_{i}-h_{0}\right)-T_{0}\left(s_{i}-s_{0}\right)$$

where the ambient temperature (*T*_0_) is set to be 298.15 K.

The exergy destruction [30,31] of each component is presented in Table 2. 

In Table 2, *T*_average_ -5 is the average temperature of the cooling medium. As for the exergy efficiency, it can be obtained by:

(5) $$\eta_{\mathrm{ex}}=\left\{ \begin{array}{l} \begin{array}{cc} \frac{W_{\mathrm{net}}}{E_{g,\mathrm{in}}-E_{g,\mathrm{mid}}} & \;\mathrm{Recuperative}\;\mathrm{cycle} \end{array}\\ \begin{array}{cc} \frac{W_{\mathrm{net}}}{E_{g,\mathrm{in}}-E_{g,\mathrm{out}}} & \;\mathrm{Split}\;\mathrm{cycle} \end{array} \end{array}\right.$$

**Figure 5:**
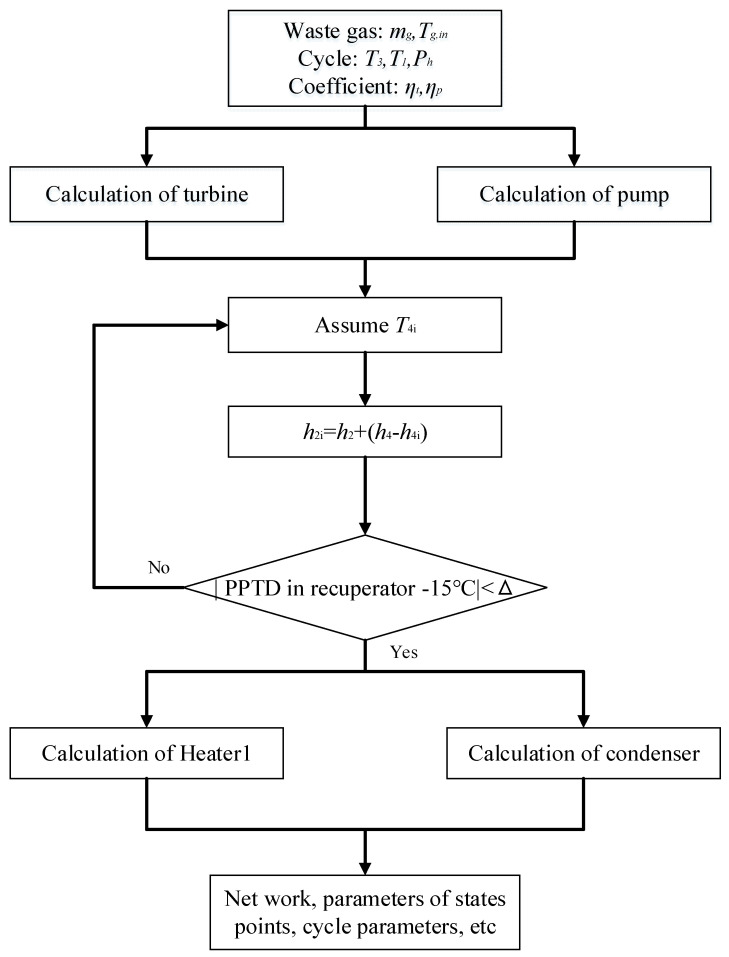
Thermodynamic calculation routine of the recuperative cycle.

**Figure 6:**
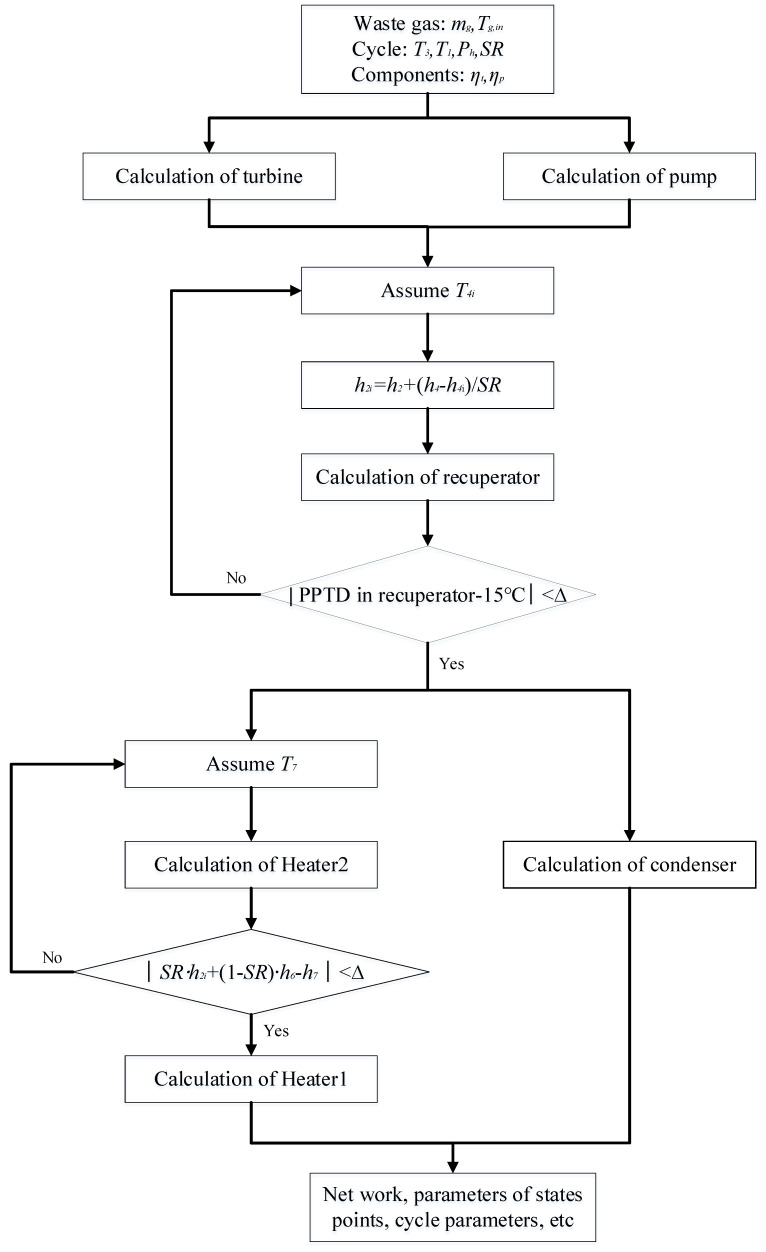
Thermodynamic calculation routine of the split cycle.

Based on the listed equations, the MATLAB platform is used for code development. The calculation flows for recuperative and split cycles are respectively given in Figure 5 and Figure 6.

### 3.2. GA Optimization

**Figure 7:**
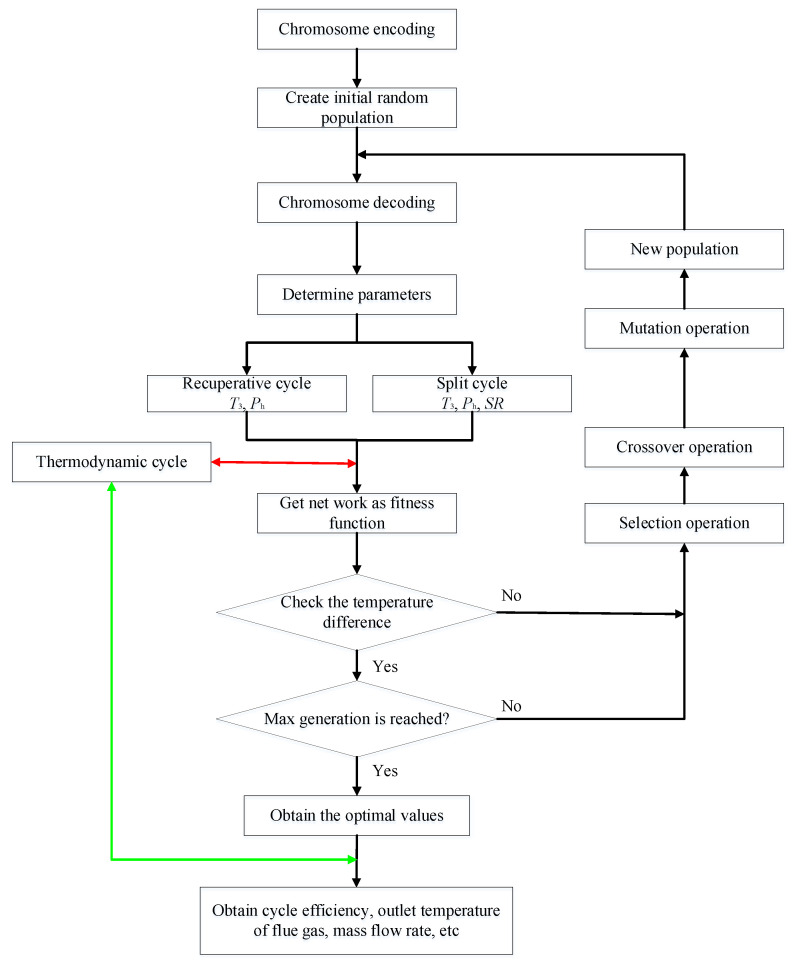
Flow diagram of GA for the system parameter optimization.

In this study, the system parameters of the two cycles are optimized by GA, due to its simplicity, versatility, and suitability for parallel processing [30,31]. Under the given ICE gas conditions, the maximum *W*_net_ is considered as the optimization objective. For the recuperative cycle, *T*_3_ and *P*_h_ are the optimization cycle variables. However, for the split cycle, *SR* is added as the third variable for performance optimization. Figure 7 gives the flowchart of the GA. The GA encodes the randomly generated chromosomes of the initial population firstly. Then the chromosome is decoded to obtain the corresponding system parameters. According to these parameters, thermodynamic calculations are conducted. The fitness function for each chromosome corresponds to the output work. Afterwards, the temperature differences in recuperator and heaters are checked to guarantee the fact that the calculated temperature difference meets the required minimum value. If not, all chromosomes will be replaced by the next population, which are propagated by choice, intersection, and mutation operators. Thereafter, *W*_net_ is obtained by recalculating the new generation. The optimization will finish when the genetic calculation is iterated to the maximum number of generations. Finally, the optimal parameters in the final population are substituted into the thermodynamic calculations to obtain the system efficiency and other parameters. In addition, the population size is programmed to 100 and the maximum number of generations is stated as 80.

## 4. Cycle conditions and Organic Fluid Selections

The working conditions of a six-cylinder engine are enumerated in Table 3. The outlet temperature and mass flow rate of the exhaust gas are 466 °C and 0.278 kg/s, respectively [23]. As for the gas components, due to the low contents of nitrogen oxides and sulfides, only CO_2_, O_2_, H_2_O, and N_2_ are considered. The corresponding mass fractions are 9.1%, 9.3%, 7.4%, and 74.2%, respectively.

The CO_2_-based mixture is formed by CO_2_ and an organic fluid. The mixture properties are strongly dependent on the selection of the organic fluid. Thus, selecting a suitable fluid is crucial to obtain a better cycle performance of the CO_2_-based mixture. In general, environmental properties have to be considered in the fluid selection. The selected fluid should be non-toxic, non-corrosiveness and stability, and have zero ozone depletion potential (ODP) and a GWP less than 150 [32].

Considering that the flue gas temperature is as high as 466 °C, the traditional refrigerant is easy to decompose, so a high temperature working fluid is selected. Based on the above standards, five kinds of organic working fluids, namely R600, R600a, R601, R601a, and R290, are selected from the organic fluid library [33]. The physical parameters of these organic working fluids and CO_2_ are summarized in Table 4.

**Figure 8:**
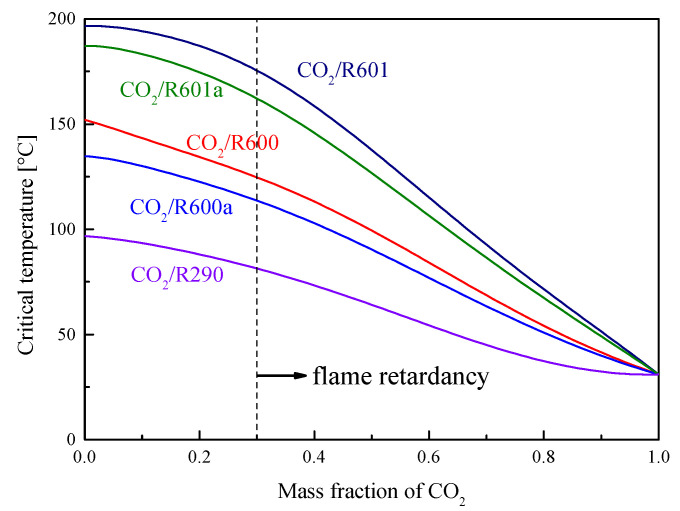
Critical temperature of mixtures under different mass fractions of CO_2_.

**Figure 9:**
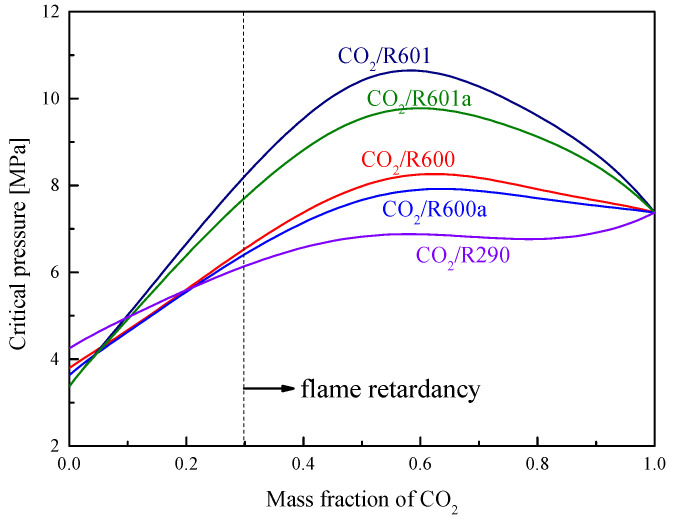
Critical pressure of mixtures under different mass fractions of CO_2_.

Figure 8 shows the critical temperatures of five mixtures at different CO_2_ fractions. It is found that the critical temperature decreases with the increase of the CO_2_ mass fraction. At the same CO_2_ fraction, the critical temperatures of five mixtures satisfy the order: R601 > R601a > R600 > R600a > R290. As for the critical pressure, Figure 9 presents the variations of these mixtures. The critical pressure firstly increases and then decreases with the increase of the CO_2_ mass fraction. When the CO_2_ fraction is larger than 0.2, the critical pressure satisfies the order: R601 > R601a > R600 > R600a > R290. In addition, it should be mentioned that the selected organic fluid is flammable. However, when CO_2_ is used as an addition to mix with the organic fluid, the flammability will be decreased with the increase of the CO_2_ fraction. According to Zabetakis’s research [19], at a CO_2_ mass fraction larger than 0.3, the mixture has exceeded the combustible range of R290. Although no similar data is available for other working fluids, the flammability of these five organic fluids is similar. Thus, in this study, it is thought that the five CO_2_-based mixtures are non-flammable, when the CO_2_ mass faction is no less than 0.3.

**Figure 10:**
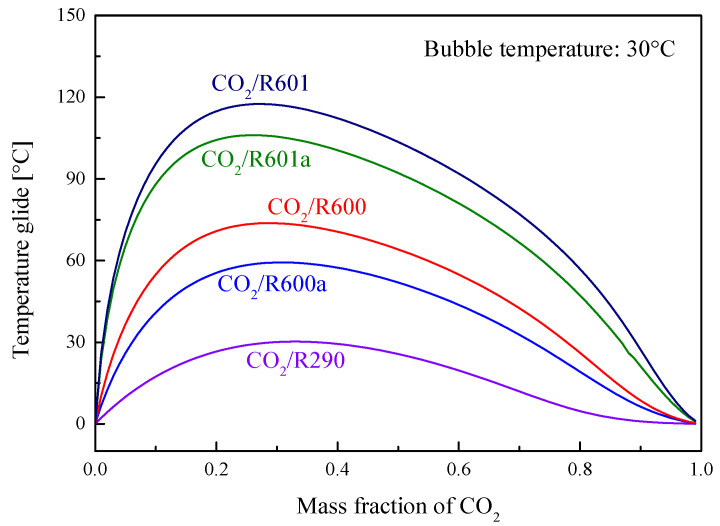
Temperature glide of mixtures under different mass fractions of CO_2_.

Figure 10 illustrates the temperature glide variation trend with CO_2_ mass fractions at the bubble temperature of 30 °C. It is obvious that when the CO_2_ mass fraction increases, the temperature glide of mixture first increases and then decreases. Among the considered mixtures, CO_2_/R290 has the lowest temperature glide, while CO_2_/R601 has the largest value. Moreover, when the CO_2_ mass fraction is around 0.3, temperature glides of these five mixtures reach the maximum value.

In addition to the employed CO_2_-based mixtures, standard design conditions are also required for the thermodynamic analysis of these two transcritcial systems. As listed in Table 5, in the thermodynamic calculation, *T*_1_, *T*_3_, and *P*_h_ are set to be 30 °C, 375 °C, and 13 MPa, respectively. For the split cycle, *SR* is assumed to be 0.7 under the basic design condition. Furthermore, isentropic efficiencies of turbine (*η*_t_) and pump (*η*_p_) are set to 0.7 and 0.8, respectively. Pinch point temperature differences (PPTDs) of the recuperator, Heater1, and Heater2 are all set at 15 °C. Meanwhile, to reduce the mixture flammability, the CO_2_ mass fraction is set at 0.3. 

To further study the thermodynamic performance of the two cycles, sensitivity analyses are conducted on *T*_3_, *P*_h_, and *T*_1_. Meanwhile, the effect of *SR* on the performance of the split cycle is also discussed. As listed in Table 5, *T*_3_ varies from 270 °C to 390 °C at an interval of 5 °C and *P*_h_ changes from 8.5 MPa to 20 MPa. In addition, the considered range of *T*_1_ is 30–90 °C. In the split cycle, *SR* varies from 0 to 1. After that, system parameters are optimized at various CO_2_ mass fractions (0.3–1) for the two cycles.

## 5. Results and Discussion

According to the conditions provided in Table 5, the recuperative cycle and the split cycle performance are derived from the established models. A synthesis of these two systems’ performances from the perspective of energy and exergy is compared. Furthermore, the effects of *T*_3_, *P*_h_, *T*_1_, and *SR* on the system performance are analyzed. On this basis, these parameters of the recuperative cycle and the split cycle are optimized with the maximum *W*_net_ as the objective. Furthermore, at different CO_2_ mass fractions, performance of these two cycles are optimized and compared. The detailed results and discussions are presented in the following subsections.

### 5.1. Cycle Analysis and Performance Comparison

Under set operating conditions, mixture characteristics at various state points are achieved for the recuperative and the split cycle, as listed in Table A1 and Table A2 of the Appendix A for CO_2_/R290(0.3/0.7). The corresponding T-s diagrams for the recuperative cycle and the split cycle are respectively presented in Figure 3 and Figure 4. Based on these state properties, energy performance of five CO_2_-based mixtures in the recuperative cycle and the split cycle are sequentially provided in Table 6 and Table 7. From Table 6, it is observed that CO_2_/R600a(0.3/0.7) has the lowest turbine outlet pressure 2.88 MPa. While the highest *W*_net_ is achieved by CO_2_/R290(0.3/0.7) and the lowest *W*_net_ is obtained by CO_2_/R601(0.3/0.7). In terms of *η*_th_, CO_2_/R290(0.3/0.7) has the highest *η*_th_ 23.74%, followed by CO_2_/R600a(0.3/ 0.7), CO_2_/R600(0.3/0.7), CO_2_/R601a(0.3/0.7) and CO_2_/R601(0.3/0.7). Thus, overall, CO_2_/R290 (0.3/0.7) has the best thermodynamic performance.

For the split cycle, *W*_net_ and *η*_th_ of CO_2_/R290(0.3/0.7) are the highest, followed by CO_2_/R600a(0.3/0.7), CO_2_/R600(0.3/0.7), and CO_2_/R601a(0.3/0.7). It should be noted that CO_2_ /R601(0.3/0.7) has the largest recuperated heat 131.49 kW, but the worst thermodynamic performance. In terms of thermodynamic performance, CO_2_/R290(0.3/0.7) is still the first choice for the split cycle. In comparison to the recuperative cycle, the split cycle possesses a higher *W*_net_, as shown in Table 7. This phenomenon is attributed by a higher mass flow rate in the split cycle. Meanwhile, the exhaust gas outlet temperature of the split cycle is lower than that of the recuperative cycle. For the considered five mixtures, the exhaust gas outlet temperature of the recuperative cycle ranges from 253.35 °C to 283.92 °C, while the split cycle has a range of 139–143.5 °C. It means that the introduction of Heater2 can deeply recover the heat from the exhaust gas for the split cycle. Thus, by constructing advanced cycle structures, the output work can be increased and fuel combustion of ICE can be reduced.

Table 8 and Table 9 respectively gives the exergy parameters of five mixtures in the recuperative cycle and split cycle under design conditions. For the exergy performance of each component, it is obvious that in the recuperative cycle, the irreversibility of the recuperator is much larger than those of other components. However, in the split cycle, the largest irreversibility is obtained by the condenser, except CO_2_/R290(0.3/0.7). As for the total irreversibility, compared with the recuperative cycle, the split cycle has a higher value, due to the addition of Heater2. For the considered five mixtures, the irreversibility of the split cycle ranges from 27.29 kW to 33.06 kW, while the recuperative cycle has a range of 18.9 kW–19.52 kW. In terms of *η*_ex_, the value of the split cycle is lower than that of the recuperative cycle for each mixture. Among the five mixtures, CO_2_/R290(0.3/0.7) has the largest efficiency in the two cycles. The corresponding values are 45.22% and 43.55%, respectively. 

### 5.2. Effects of Key System Parameters

#### 5.2.1. Effect of Turbine Inlet Temperature

**Figure 11:**
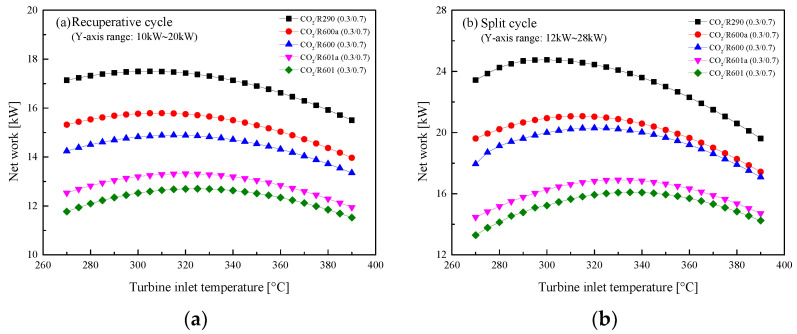
Effect of turbine inlet temperature on net work: (**a**) recuperative cycle, (**b**) split cycle.

Figure 11 illustrates the changes of *W*_net_ with *T*_3_ for the two cycles. The *T*_3_ is investigated in the range of 270 °C to 390 °C. From Figure 11a, it is found that the *W*_net_ of the recuperative cycle tends to increase and then decrease as *T*_3_ increases. This phenomenon can be interpreted based on the fact that with the increase of *T*_3_, the mass flow of mixtures gradually decreases. Under a lower *T*_3_, the variation of *W*_net_ is mainly affected by the enthalpy difference; while under a higher *T*_3_, the change of *W*_net_ depends on the mass flow rate. As for the considered five mixtures, CO_2_/R290(0.3/0.7) has the largest *W*_net_ in the recuperative cycle, followed by CO_2_/R600a(0.3/0.7), CO_2_/R600(0.3/0.7), CO_2_/R601a(0.3/0.7) and CO_2_/R601(0.3/ 0.7). As for the split cycle, similar trends of these mixtures are observed, and *W*_net_ satisfies the order: CO_2_/R601(0.3/0.7) > CO_2_/R601a(0.3/0.7) > CO_2_/R600(0.3/0.7) > CO_2_/R600a(0.3/0.7) > CO_2_ / R290(0.3/0.7). Meanwhile, for the same mixture, the split cycle has a larger *W*_net_ than that of the recuperative cycle. Furthermore, in these two cycles, when the organic fluids are isomers, the corresponding CO_2_ mixtures perform a similar performance, such as CO_2_/R600a(0.3/0.7) and CO_2_/R600(0.3/0.7).

**Figure 12:**
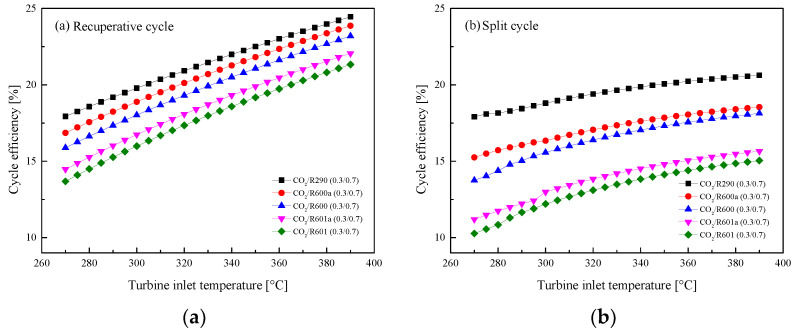
Effect of turbine inlet temperature on cycle efficiency: (**a**) recuperative cycle, (**b**) split cycle.

**Figure 13:**
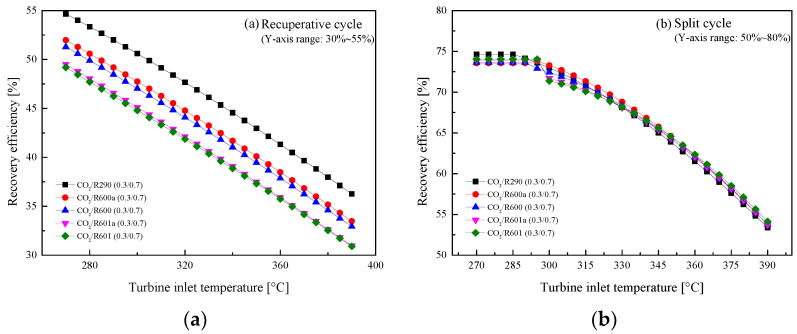
Effect of turbine inlet temperature on recovery efficiency: (**a**) recuperative cycle, (**b**) split cycle.

The variations of *η*_th_ with *T*_3_ are shown in Figure 12. The figure presents that *η*_th_ naturally increases with the increase of *T*_3_ in the two cycles. Although the *W*_net_ of the split cycle is greater than that of the recuperative cycle, the split *η*_th_ is significantly lower than that of the recuperative cycle. This is due to the fact that the added Heater2 absorbs more heat from the exhaust gas in the split cycle. Among the considered five mixtures, CO_2_/R290(0.3/0.7) shows the best *η*_th_, followed by CO_2_/R600a(0.3/0.7), CO_2_/R600(0.3/0.7), CO_2_/R601a(0.3/0.7), and CO_2_/R601(0.3/0.7). The effects of *T*_3_ on *η*_r_ are presented in Figure 13. It can be seen that the heat *η*_r_ decreases as *T*_3_ increases. Meanwhile, being different with efficiency curves of the recuperative cycle, the curves of the split cycle among the five mixtures are more closed with each other.

#### 5.2.2. Effect of Turbine Inlet Pressure

**Figure 14:**
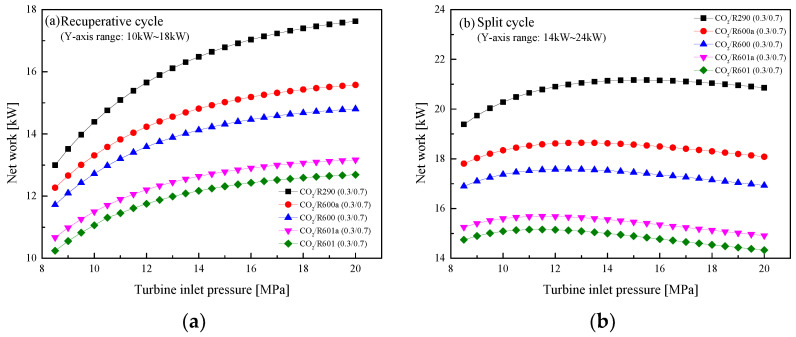
Effect of turbine inlet pressure on net work: (**a**) recuperative cycle, (**b**) split cycle.

**Figure 15:**
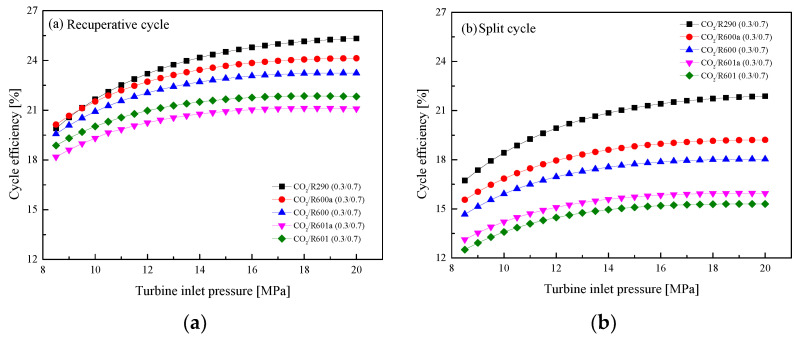
Effect of turbine inlet pressure on cycle efficiency: (**a**) recuperative cycle, (**b**) split cycle.

In this section, the effect of *P*_h_ on the system performance is revealed by varying the pressure settings from 8.5 MPa to 20 MPa in the step of 0.5MPa. Figure 14 shows the effects of *P*_h_ on *W*_net_ in the recuperative cycle and the split cycle. It can be observed from Figure 14a that in the recuperative cycle, *W*_net_ increases as *P*_h_ increases, while the growth speed gradually slows down at around 14 MPa. By comparison, the *W*_net_ of the five mixtures is ordered by CO_2_/R290(0.3/0.7) > CO_2_/R600a(0.3/0.7) > CO_2_/R600(0.3/0.7) > CO_2_/R601a(0.3/0.7) > CO_2_/R60 1(0.3/0.7). As for the split cycle, when the pressure gradually increases, *W*_net_ increases first and then decreases, as illustrated in Figure 14b. For the performance comparison, the split cycle has a larger work than the recuperative cycle for the same mixture. Taking CO_2_/R600a(0.3/0.7) as an example, when *P*_h_ is 16 MPa, the *W*_net_ of the split cycle is 18.5 kW and that of the recuperative cycle is 15.42 kW. This is mainly due to a large mass flow rate of the mixture in the split cycle. The variations of *η*_th_ with *P*_h_ are shown in Figure 15. It can be seen that with the increase of *P*_h_, *η*_th_ first increases and then remains stable in the recuperative cycle and the split cycle. Different from the comparison of *W*_net_, the *η*_th_ of the recuperative cycle is higher than that of the split cycle. This can be explained by the absorption of more waste heat in the split cycle.

**Figure 16:**
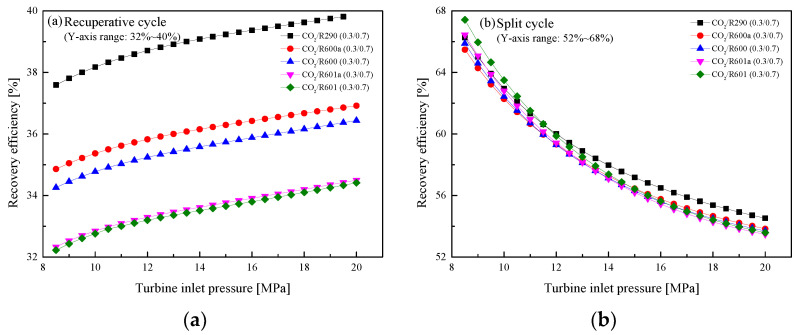
Effect of turbine inlet pressure on recovery efficiency: (**a**) recuperative cycle, (**b**) split cycle.

Figure 16a,b show *η*_r_ curves of two cycles at different *P*_h_. It is clearly seen from Figure 16a that in the recuperative cycle, *η*_r_ increases rapidly with the increase of *P*_h_. This is because the heat absorbed by Heater1 from the exhaust gas gradually increases with the increase of *P*_h_. On the contrary, *η*_r_ of the split cycle decreases with the increase of *P*_h_, as shown in Figure 16b. This can be explained by the decrease of mass flow rate in the split cycle. Even so, compared with the recuperative cycle, *η*_r_ of the split cycle is still higher, and this increase of efficiency is due to the addition of Heater2, which increases the total heat absorption of the system.

#### 5.2.3. Effect of Condensation Temperature

**Figure 17:**
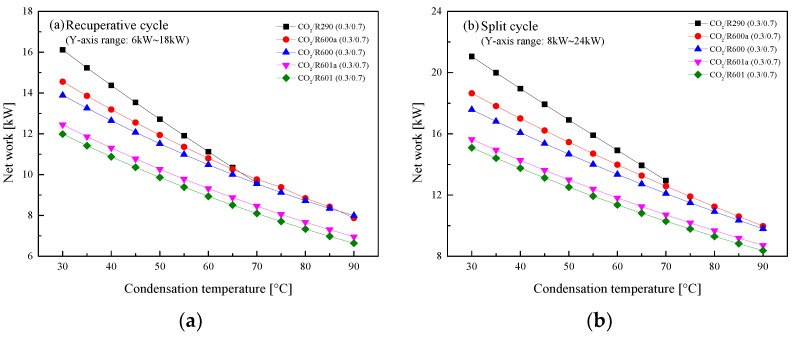
Effect of condensation temperature on net work: (**a**) recuperative cycle, (**b**) split cycle.

In general, there are three different cooling methods for fluid condensation in ICE [27]. The obtained *T*_1_ ranges from 30 °C to 90 °C. Thus, the temperature effects on the performance of the recuperative cycle and the split cycle are analyzed. Special care should be given CO_2_/R290(0.3/0.7). Due to the limit of critical temperature, the maximum *T*_1_ is set to be 70 °C to guarantee the subcritical condensation of the mixture. Figure 17 presents the variations of the *W*_net_ with *T*_1_ in the two cycles. It is clear that the *W*_net_ of the recuperative cycle and the split cycle naturally decrease as *T*_1_ increases. Taking CO_2_/R290(0.3/0.7) as an example, the *W*_net_ of the split cycle decreases about 4 kW and that of the recuperative cycle decreases about 3 kW for every 20 °C rises of *T*_1_.

**Figure 18:**
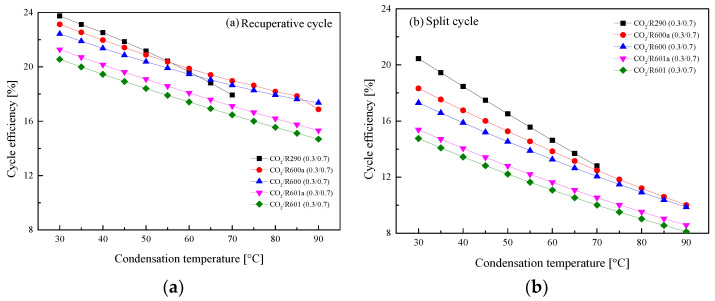
Effect of condensation temperature on cycle efficiency: (**a**) recuperative cycle, (**b**) split cycle.

Figure 18 shows that *η*_th_ of two cycles decrease with the increase of *T*_1_. It is worth noting that in the recuperative cycle, when *T*_1_ is lower than 55 °C, the *W*_net_ of the considered mixtures satisfy the following order: CO_2_/R290(0.3/0.7) > CO_2_/R600a(0.3/0.7) > CO_2_/R600 (0.3/0.7) > CO_2_/R601a(0.3/0.7) > CO_2_/R601(0.3/0.7). Meanwhile, for the considered five mixtures in the split cycle, CO_2_/R290(0.3/0.7) has the largest *η*_th_ and CO_2_/R601(0.3/0.7) has the lowest efficiency when the *T*_1_ range is 30–70 °C. In addition, for every 20 °C increase of *T*_1_, *η*_th_ of CO_2_/R290(0.3/0.7) in the split cycle decreases about 4%, while that of recuperative cycle is decreased by about 3%.

**Figure 19:**
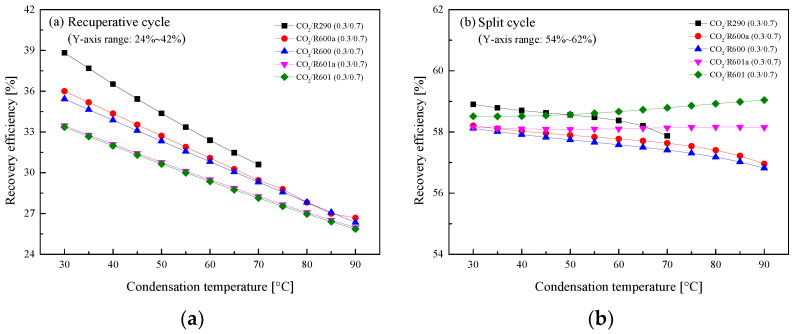
Effect of condensation temperature on recovery efficiency: (**a**) recuperative cycle, (**b**) split cycle.

The influences of *T*_1_ on *η*_r_ for the recuperative cycle and the split cycle are illustrated in Figure 19. It can be found that *η*_r_ decreases with the increase of *T*_1_ in the recuperative cycle from Figure 19a. It can be explained by the fact that the increase of *T*_1_ will enlarge the recuperated heat, thus decreasing the absorbed heat in Heater1. When *T*_1_ increases 20°C, *η*_r_ of CO_2_/R290(0.3/0.7) in the recuperative cycle decreases about 4%. However, for the split cycle, the increase of *T*_1_ affects *η*_r_ slightly for different mixture fluids, as shown in Figure 19b. This is because that although the heat of Heater1 decreases, the heat absorbed in Heater2 continues to increase. Therefore, the total heat absorption of the split cycle varies little. Taking CO_2/_R290(0.3/0.7) as an example, *T*_1_ increases from 30 °C to 70 °C, and *η*_r_ decreases from 59% to 58%.

#### 5.2.4. Effect of Split Ratio

In the above analysis, *SR* is set to 0.7 for the split cycle. However, in practical engineering, the *SR* can be manually controlled. When the value is set to 1, it represents the recuperative cycle. Meanwhile, at a *SR* of 0, it means that the split cycle becomes a basic transcritical cycle. Therefore, the effect of *SR* on the split cycle performance is worth investigating.

**Figure 20:**
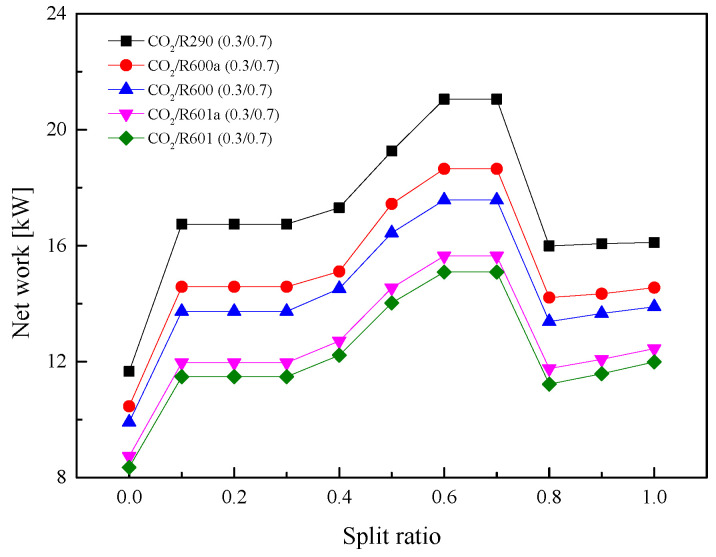
Effect of split ratio on net work in the split cycle.

Figure 20 shows the variation curves of *W*_net_ in split ratio within the range of 0–1. It is obvious from the figure that with the increase of *SR*, *W*_net_ firstly increases and then decreases. There exists a maximal value for each mixture. This is because that at a small *SR*, the split cycle is close to a simple transcritical cycle, thus producing a lower *W*_net_. Meanwhile, when *SR* is close to 1, the split cycle is more similar to the recuperative cycle, and the advantage of the split is weakened. It should be noted that when *SR* is larger than 0.8, there exists a little rise of *W*_net_. Similarly, for the five mixtures, *W*_net_ satisfies the order: CO_2_/R290(0.3/0.7) > CO_2_/R600a(0.3/0.7) >CO_2_/R600(0.3/0.7)> CO_2_/R601a(0.3/0.7) > CO_2_/R601(0.3/0.7).

**Figure 21:**
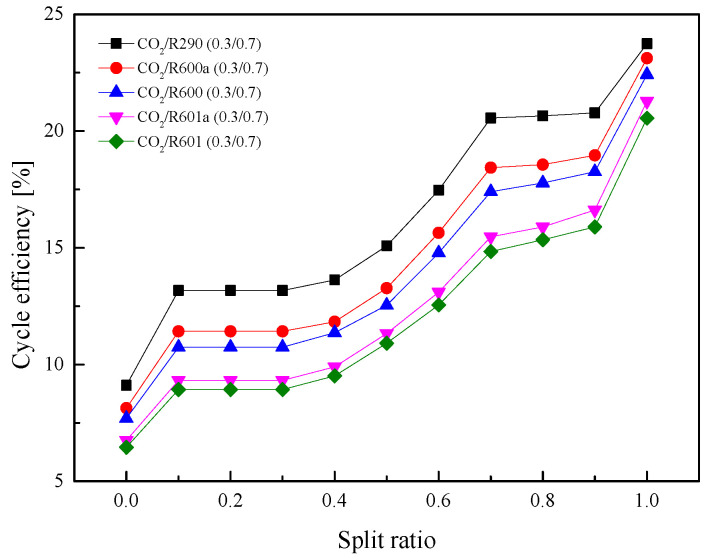
Effect of split ratio on cycle efficiency in the split cycle.

**Figure 22:**
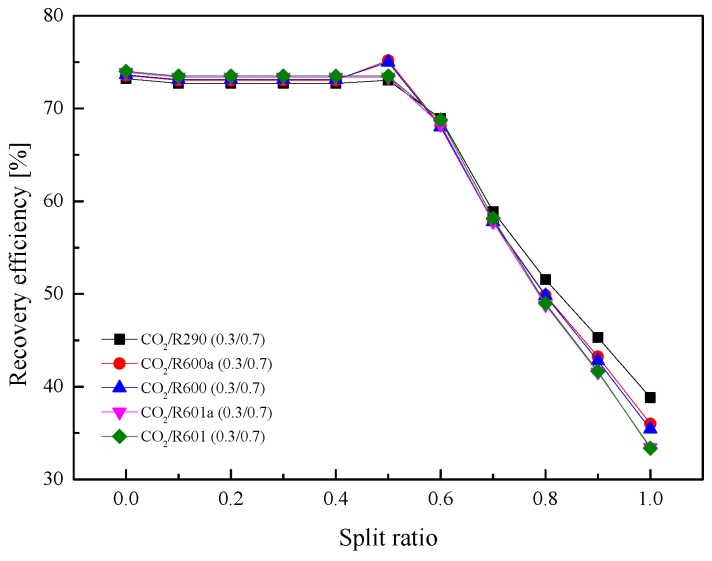
Effect of split ratio on recovery efficiency in the split cycle.

As for the *η*_th_ in Figure 21, when *SR* gradually increases, *η*_th_ of five mixtures increases continuously. This is because a higher ratio led to a larger recuperated heat. The total heat absorbed in Heater1 and Heater2 continuously decreases. It can be observed that when *SR* is 0, *η*_th_ is lower than 10%. While as *SR* is 1, *η*_th_ is higher than 20%. Figure 22 shows the variations of *η*_r_ under different *SR*. As *SR* increases, *η*_r_ at first barely changes and then decreases considerably. More working fluid enters Heater2 when *SR* is relatively small, so *η*_r_ is high. After *SR* increases to a certain extent, the cycle is close to the recuperative cycle, so *η*_r_ is reduced.

### 5.3. Parametric Optimization

Based on the above analysis, it can be concluded that *T*_3_, *P*_h_, *T*_1_, and *SR* have different effects on cycle performance. However, due to the fact that *T*_1_ is determined by the cooling method, *T*_1_ here is set to 30 °C. For *W*_net_ optimization of the recuperative cycle, *T*_3_ and *P*_h_ are set in the range of 240–400 °C and 8.5 MPa–20 MPa, respectively. As for the split cycle, *SR* ranges from 0 to 1. On this basis, the optimization results for the recuperative cycle and the split cycle are provided in Table 10 and Table 11, respectively. 

For the recuperative cycle, Table 10 shows that the optimized *W*_net_ is substantially increased, compared to *W*_net_ under the basic operating condition. For instance, *W*_net_ of CO_2_ /R290(0.3/0.7) under basic operating conditions is 16.11 kW, while the optimized *W*_net_ is 18.33 kW. The increase of *W*_net_ reaches up to 13.78%. Among the five mixtures, a *W*_net_ of CO_2_/R290(0.3/0.7) is the largest and that of CO_2_/R601(0.3/0.7) is the smallest. As for the split cycle, performance is also greatly improved. From Table 11, for CO_2_/R290(0.3/0.7), compared to *W*_net_ of 21.05 kW at basic operating conditions, the optimized *W*_net_ of 23.37 kW increases 11.02%. Compared with the optimization results of the recuperative cycle, the split cycle has a larger *W*_net_ and a higher *η*_r_ for the considered mixtures.

### 5.4. Performance Comparison at Different CO_2_ Mass Fractions

Considering that the mixture properties change considerably with the CO_2_ mass fraction, performance of the two cycles are further obtained at different fractions. To avoid the mixture flammability, CO_2_ mass fraction ranges from 0.3 to 1.0. In this range, performance comparisons are respectively conducted under design and optimization conditions. The details are presented in the following subsections.

#### 5.4.1. Design Conditions

**Figure 23:**
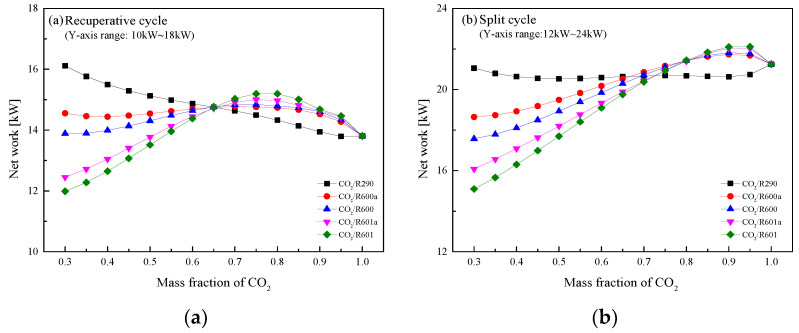
Net work of CO_2_-based mixtures at different CO_2_ mass fractions: (**a**) recuperative cycle, (**b**) split cycle.

Figure 23 presents the variations of *W*_net_ with the CO_2_ mass fraction for the recuperative and split cycles under design conditions. In Figure 23a for the recuperative cycle, it is clear that except CO_2_/R290, with the increase of CO_2_ mass fraction, the *W*_net_ of the other four mixtures first increase and then decrease. This phenomenon can be explained by the temperature glides. At a lower CO_2_ mass fraction, the temperature glide of the five mixtures is large and the *W*_net_ is relatively small. However, with the increase of a CO_2_ mass fraction, the temperature glide gradually decreases and the *W*_net_ of the mixtures gradually increases. It should be noted that the *W*_net_ of the five mixtures are equal with each other at the CO_2_ mass fraction 0.65. When CO_2_ mass fraction is lower than 0.65, CO_2_/R290 exhibits the highest *W*_net_, followed by CO_2_/R600a, CO_2_/R600, CO_2_/R601a, and CO_2_/R601. However, when the CO_2_ mass fraction is greater than 0.65, a small difference exists for the *W*_net_ of each mixture. As for the split cycle, the variation trend of *W*_net_ is similar to that in the recuperative cycle. Being different with the recuperative cycle, the split cycle has an equal *W*_net_ for different mixtures at the CO_2_ fraction 0.7. When the fraction is less than 0.7, the highest work is still obtained by CO_2_/R290. While, the value of net work of CO_2_/R290 is the lowest when the fraction is higher than 0.7. In terms of the *W*_net_ comparison between the two cycles, the split cycle always has a larger work than that of the recuperative cycle.

**Figure 24:**
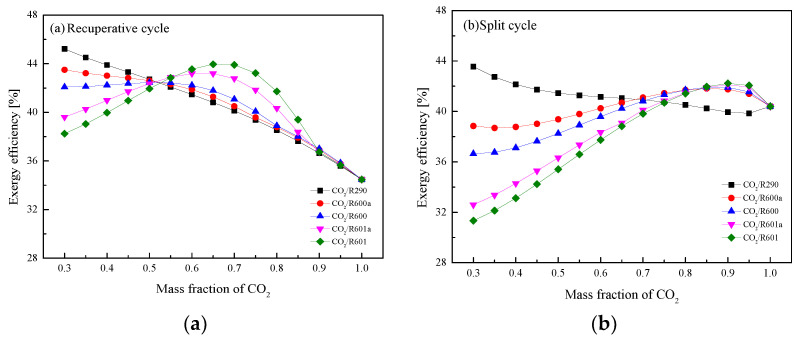
Exergy efficiency of CO_2_-based mixtures at different CO_2_ mass fractions: (**a**) recuperative cycle, (**b**) split cycle.

At different CO_2_ mass fractions, *η*_ex_ of the two cycles are provided in Figure 24. It can be observed that *η*_ex_ has similar curves with *W*_net_, in terms of the variation trends and curve distributions. For the recuperative cycle, the minimum *η*_ex_ 34.46% is obtained by pure CO_2_, while the maximum efficiencies of these mixtures are obtained at different fractions. Among the five working fluids, Figure 24a depicts that when the mass fraction of CO_2_ is 0.3, CO_2_/R290 has the highest *η*_ex_ of 45.22%. It should be noted that the equal efficiencies of different mixtures are obtained at the CO_2_ mass fraction 0.5. For the split cycle in Figure 24b, the maximum efficiency 43.55% among the five mixtures is obtained by CO_2_/R290 (0.3/0.7). Meanwhile, mixtures have the minimum efficiency at different mass fractions. For the considered five mixtures, the lowest efficiency are 39.85%, 38.69%, 36.65%, 32.72% and 31.45% for CO_2_/R290(0.95/0.05), CO_2_/R600a(0.35/ 0.65), CO_2_/R600(0.3/0.7), CO_2_/R601a(0.3/0.7) and CO_2_/R601(0.3/0.7), respectively.

**Figure 25:**
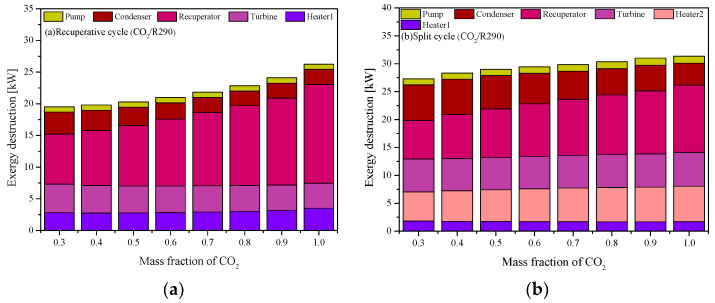
Exergy destruction of CO_2_-based mixtures at different CO_2_ mass fractions: (**a**) recuperative cycle, (**b**) split cycle.

Figure 25 shows the variation trends of the component irreversibility with the CO_2_ mass fraction. For the two cycles, with the mass fraction of CO_2_ increases, the irreversibility of the recuperator increases significantly. When the mass fraction of CO_2_ is low, the mixture has a better thermal match in the recuperator. However, opposite variations are observed for turbine in the recuperative cycle. Furthermore, for the split cycle in Figure 25b, with the increase of CO_2_ mass fraction, the irreversibility in Heater2 increases. Due to this, the total irreversibility of the split cycle is always larger than that of the recuperative cycle.

#### 5.4.2. Optimization Conditions

To further compare the system performance, the maximum *W*_net_ at different CO_2_ mass fractions is obtained by parametric optimization. Similarly, in the optimization process, *T*_1_ is set to 30°C. Considering the fact that the employed mixture has the highest critical pressure of 10.64 MPa for CO_2_/R601, the *P*_h_ ranges from 11 MPa to 20 MPa. For *T*_3_, the range is 240 °C–400 °C. Furthermore, in the split cycle, *SR* varies from 0 to 1.

**Figure 26:**
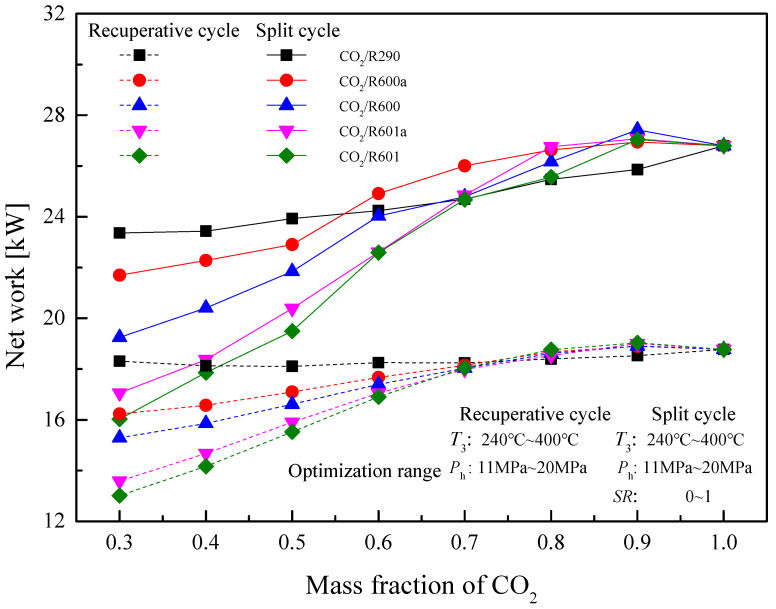
Optimal net work of recuperative and split cycles at different CO_2_ mass fractions.

Figure 26 shows the optimal *W*_net_ of recuperative and split cycles at different CO_2_ mass fractions. It can be noted that the *W*_net_ of two cycles increases with the increase of the CO_2_ mass fraction. Among the considered five mixtures, when the mass fraction of CO_2_ is lower than 0.6, the *W*_net_ of CO_2_/R290 is the largest in the split cycle. Meanwhile, it is clear that at the CO_2_ mass fraction of 0.9, CO_2_/R600 obtains the maximum *W*_net_. For the recuperative cycle, as the CO_2_ mass fraction is lower than 0.7, the *W*_net_ of five mixtures satisfies the order: CO_2_/R290 > CO_2_/R600a > CO_2_/R600 > CO_2_/R601a > CO_2_/R601. Through the performance comparison, the *W*_net_ of the split cycle is greater than that of the recuperative cycle at different CO_2_ mass fractions for five mixtures. Moreover, the specific optimization parameters of the recuperative cycle and the split cycle are listed in Table 12 and Table 13, respectively.

## 6. Conclusions

In this paper, two transcritical systems, namely the recuperative cycle and the split cycle, are employed to recover the waste heat of ICE. Five CO_2_-based mixtures namely R290, R600a, R600, R601a, and R601 are used. Based on the thermodynamic calculations, cycle performance is analyzed and compared. Key parameters including turbine inlet temperature, turbine inlet pressure, condensation temperature, and split ratio are investigated to reveal the effects on the system performance. On this basis, these key parameters are optimized to achieve the maximum net work in two cycles. Meanwhile, cycle performance of mixtures at different CO_2_ mass fractions are compared under design and optimization conditions. From the above results, the main conclusions are drawn as follows:

Under design conditions, in the recuperative cycle, the maximum cycle efficiency of 23.74% is obtained by CO_2_/R290(0.3/0.7). In addition, the largest net work of 21.05 kW in the split cycle is gained by CO_2_/R290(0.3/0.7). Compared with the recuperative cycle, the net work and recovery efficiency of the split cycle are much higher.

With the increase of turbine inlet temperature, the net works of the two cycles tend to increase firstly and then decrease, while the cycle efficiency continuously increases and recovery efficiency decreases greatly. Furthermore, as the turbine inlet pressure increases, the net work of the recuperative cycle gradually increases, whereas the net work of the split cycle firstly increases and then decreases. For the effect of the split ratio, as the split ratio increases, the net work of the split cycle first increases and then decreases.

Under design conditions, as the CO_2_ mass fraction increases, the net work of mixtures except CO_2_/R290 shows a trend of first increasing and then decreasing in the recuperative cycle and the split cycle. However, under optimization conditions, the maximum net work increases continuously. The maximum net work of the split cycle is obtained by CO_2_/R600(0.9/0.1) and is 27.43 kW.

## Figures and Tables

**Table 1:** Energy equation of each component for the recuperative cycle and the split cycle.

Components	Recuperative Cycle	Split Cycle
Pump	$W_{p}=m_{f}\left(h_{2}-h_{1}\right)=m_{f}\left(h_{2s}-h_{1}\right)/\eta_{p}$	
Turbine	$W_{t}=m_{f}\left(h_{3}-h_{4}\right)=m_{f}\left(h_{3}-h_{4s}\right)\eta_{t}$	
Condenser	$Q_{\mathrm{con}\;}=m_{f}\left(h_{4i}-h_{1}\right)$	
Heater1	$Q_{\mathrm{Heater}1\;}=m_{f}\left(h_{3}-h_{2i}\right)=m_{g}C_{p,g}\left(T_{g,\mathrm{in}\;}-T_{g,\mathrm{mid}}\right)$	
Recuperator	QRe=mf(h4−h4i)	
Heater2	---	$Q_{\mathrm{Heater}2}=m_{f}(1-SR)\left(h_{6}-h_{5}\right)$

**Table 2:** Exergy destruction of each component for the recuperative cycle and the split cycle.

Components	Recuperative Cycle	Split Cycle
Pump	$I_{p}=m_{f}\left(e_{1}-e_{2}\right)+W_{p}$	
Turbine	$I_{t}=m_{f}\left(e_{3}-e_{4}\right)-W_{t}$	
Condenser	$I_{\mathrm{con}\;}=m_{f}\left(e_{4i}-e_{1}\right)-Q_{\mathrm{con}}\left[1-T_{0}/\left(T_{\mathrm{average}}\text{-}5\right)\right]$	
Heater1	$I_{\mathrm{Heater}1\;}=m_{f}\left(e_{2i}-e_{3}\right)+E_{g,\mathrm{in}}-E_{g,\mathrm{mid}}$	
Recuperator	IRe=mf(e2−e2i+e4−e4i)	IRe=mf⋅SR(e2−e2i)+mf(e4−e4i)
Heater2	---	$I_{\mathrm{Heater}2\;}=m_{f}(1-SR)\left(e_{5}-e_{6}\right)+E_{g,\mathrm{mid}}-E_{g,\mathrm{out}}$

**Table 3:** Main parameters and exhaust gas components of ICE.

Parameters	Values	Exhaust Gas Components	Values
Fuel combustion energy (kW)	561.61	CO_2_ (%)	9.1
Rated power (kW)	236.50	O_2_ (%)	9.3
Rated speed (rpm)	1694	H_2_O (%)	7.4
Rated torque (N·m)	1333	N_2_ (%)	74.2
Temperature of exhaust gas (°C)	466		
Mass flow of exhaust gas (kg/s)	0.278		
Exhaust gas pressure (MPa)	0.10		

**Table 4:** Thermodynamic properties of the selected working fluids.

Fluids	Molecular Mass (g/mol)	*T*_b_ (°C)	*T*_c_ (°C)	*P*_c_ (MPa)
CO_2_	44.01	−78.4	31.1	7.38
R290	44.1	−42.1	96.7	4.25
R600a	58.12	−11.7	134..7	3.63
R600	58.12	−0.5	152.0	3.80
R601a	72.15	27.83	187.2	3.38
R601	72.15	36.06	196.55	3.37

**Table 5:** Standard design conditions of the two transcritical systems.

Design Parameters	Set Value	Range of Variation
Condensation bubble temperature (°C)	30	30–90
Turbine inlet temperature (°C)	375	270–390
Turbine inlet pressure (MPa)	13	8.5–20
Ambient temperature (°C)	25	—
Ambient pressure (MPa)	0.1	—
PPTD in Heater1 (°C)	15	—
PPTD in Heater2 (°C)	15	—
PPTD in recuperator (°C)	15	—
Pump efficiency	0.8	—
Turbine efficiency	0.7	—
Split ratio	0.7	0–1
Mass fraction of CO_2_	0.3	0.3–1

**Table 6:** Energy performance of five mixtures in the recuperative cycle under design conditions.

Parameters	CO_2_/R290	CO_2_/R601a	CO_2_/R601	CO_2_/R600a	CO_2_/R600
Mass fraction of CO_2_	0.3	0.3	0.3	0.3	0.3
*T*_mid_ (°C)	253.35	283.34	283.92	269.16	272.39
*P*_L_ (MPa)	3.27	2.95	3.06	2.88	3.00
*m*_f_ (kg/s)	0.19	0.20	0.20	0.20	0.20
*Q*_Heater1_ (kW)	67.86	58.51	58.33	62.94	61.93
*Q*_Re_ (kW)	109.07	125.86	127.40	116.08	118.54
*W*_t_ (kW)	20.45	16.34	15.68	18.76	18.16
*W*_p_ (kW)	4.34	3.89	3.69	4.21	4.28
*W*_net_ (kW)	16.11	12.45	11.99	14.55	13.89
*η*_th_ (%)	23.74	21.28	20.55	23.12	22.42
*η*_r_ (%)	38.82	33.46	33.36	36.00	35.42

**Table 7:** Energy performance of five mixtures in the split cycle under design conditions.

Parameters	CO_2_/R290	CO_2_/R601a	CO_2_/R601	CO_2_/R600a	CO_2_/R600
Mass fraction of CO_2_	0.3	0.3	0.3	0.3	0.3
*T*_out_ (°C)	139.00	143.29	141.22	143.00	143.50
*T*_mid_ (°C)	316.68	323.12	323.25	319.67	319.05
*P*_L_ (MPa)	3.27	2.95	3.06	2.88	3.00
*m*_f_ (kg/s)	0.25	0.25	0.25	0.25	0.25
*Q*_Heater1_ (kW)	48.03	45.99	45.95	47.08	47.28
*Q*_Heater2_ (kW)	54.95	55.68	56.35	54.68	54.33
*Q*_Re_ (kW)	128.22	129.92	131.49	127.59	126.77
*W*_t_ (kW)	26.72	20.53	19.74	24.04	22.99
*W*_p_ (kW)	5.67	4.89	4.65	5.39	5.41
*W*_net_ (kW)	21.05	15.64	15.09	18.65	17.57
*η*_th_ (%)	20.44	15.38	14.75	18.32	17.29
*η*_r_ (%)	58.90	58.15	58.51	58.21	58.12

**Table 8:** Exergy performance of five mixtures in the recuperative cycle under design conditions.

Parameters	CO_2_/R290	CO_2_/R600a	CO_2_/R600	CO_2_/R601a	CO_2_/R601
Mass fraction of CO_2_	0.3	0.3	0.3	0.3	0.3
*I*_Heater1_ (kW)	2.80	2.55	2.50	2.33	2.33
*I*_t_ (kW)	4.50	4.10	3.98	3.54	3.40
*I*_Re_ (kW)	7.91	6.90	6.82	7.30	7.66
*I*_con_ (kW)	3.48	4.54	4.99	5.06	5.26
*I*_p_ (kW)	0.83	0.81	0.82	0.75	0.71
*I*_total_ (kW)	19.52	18.90	19.11	18.98	19.36
*η*_ex_ (%)	45.22	43.50	42.08	39.61	38.24

**Table 9:** Exergy performance of five mixtures in the split cycle under design conditions.

Parameters	CO_2_/R290	CO_2_/R600a	CO_2_/R600	CO_2_/R601a	CO_2_/R601
Mass fraction of CO_2_	0.3	0.3	0.3	0.3	0.3
*I*_Heater1_ (kW)	1.81	1.77	1.78	1.73	1.73
*I*_Heater2_ (kW)	5.22	5.00	4.94	4.90	4.87
*I*_t_ (kW)	5.89	5.26	5.03	4.45	4.28
*I*_Re_ (kW)	6.94	6.94	6.87	7.69	7.96
*I*_con_ (kW)	6.35	9.35	10.73	12.63	13.33
*I*_p_ (kW)	1.08	1.03	1.04	0.94	0.90
*I*_total_ (kW)	27.29	29.35	30.38	32.34	33.06
*η*_ex_ (%)	43.55	38.85	36.65	32.60	31.34

**Table 10:** Optimized cycle parameters of the recuperative cycle.

Parameters	CO_2_/R290	CO_2_/R600a	CO_2_/R600	CO_2_/R601a	CO_2_/R601
Mass fraction of CO_2_	0.3	0.3	0.3	0.3	0.3
*T*_1_ (°C)	30	30	30	30	30
*T*_3_ (°C)	322.94	325.57	333.80	334.03	341.40
*P*_h_ (MPa)	19.51	19.47	19.23	19.42	19.42
*T*_mid_ (°C)	204.67	221.72	230.05	247.47	248.24
*P*_L_ (MPa)	3.27	2.88	3.00	2.95	3.06
*m*_f_ (kg/s)	0.23	0.24	0.23	0.23	0.23
*Q*_Heater1_ (kW)	82.91	77.66	75.09	69.69	69.45
*Q*_Re_ (kW)	87.62	98.24	101.85	110.86	112.00
*W*_t_ (kW)	26.76	24.42	23.34	20.79	19.88
*W*_p_ (kW)	8.43	8.18	8.06	7.21	6.86
*W*_net_ (kW)	18.33	16.24	15.29	13.58	13.01
*η*_th_ (%)	22.10	20.91	20.36	19.49	18.73
*η*_r_ (%)	47.42	44.42	42.95	39.86	39.72

**Table 11:** Optimized cycle parameters of the split cycle.

Parameters	CO_2_/R290	CO_2_/R600a	CO_2_/R600	CO_2_/R601a	CO_2_/R601
Mass fraction of CO_2_	0.3	0.3	0.3	0.3	0.3
*T*_1_ (°C)	30	30	30	30	30
*T*_3_ (°C)	344.68	309.09	320.86	330.29	334.36
*P*_h_ (MPa)	12.01	11.47	13.31	12.51	13.2
*SR*	0.67	0.78	0.66	0.74	0.63
*T*_mid_ (°C)	289.47	256.53	261.17	277.49	279.40
*T*_out_ (°C)	62.83	100.57	54.18	107.44	51.40
*P*_L_ (MPa)	3.27	2.88	3.00	2.95	3.06
*m*_f_ (kg/s)	0.31	0.38	0.33	0.33	0.31
*Q*_Heater1_ (kW)	56.59	66.88	65.43	60.34	59.74
*Q*_Heater2_ (kW)	69.37	47.73	63.12	52.20	69.64
*Q*_Re_ (kW)	140.84	169.23	122.53	148.56	121.00
*W*_t_ (kW)	29.78	28.63	26.55	23.08	21.84
*W*_p_ (kW)	6.40	6.93	7.30	6.03	5.81
*W*_net_ (kW)	23.37	21.70	19.25	17.06	16.03
*η*_th_ (%)	18.56	18.93	14.97	15.16	12.39
*η*_r_ (%)	72.04	65.55	73.53	64.37	74.00

**Table 12:** Optimal cycle parameters of the recuperative cycle at different CO_2_ mass fractions.

Fluids	Parameters	Mass Fraction of CO_2_							
		0.30	0.40	0.50	0.60	0.70	0.80	0.90	1.00
CO_2_/R290	*T*_3_ (°C)	322.94	320.69	320.45	324.33	323.66	332.79	339.31	353.74
	*P*_h_ (MPa)	19.51	19.34	19.09	19.68	19.08	20.00	19.70	19.86
	*W*_net_ (kW)	18.33	18.13	18.11	18.25	18.24	18.40	18.33	18.77
CO_2_/R600a	*T*_3_ (°C)	325.57	324.15	320.56	321.40	323.95	332.19	339.29	353.74
	*P*_h_ (MPa)	19.47	19.17	18.96	19.17	19.23	19.86	19.67	19.86
	*W*_net_ (kW)	16.24	16.57	17.10	17.67	18.15	18.68	18.90	18.77
CO_2_/R600	*T*_3_ (°C)	333.80	329.38	324.45	323.27	326.42	333.90	333.90	353.74
	*P*_h_ (MPa)	19.23	19.38	18.91	19.07	19.22	19.87	19.66	19.86
	*W*_net_ (kW)	15.29	15.86	16.61	17.39	18.04	18.62	18.90	18.77
CO_2_/R601a	*T*_3_ (°C)	334.03	331.62	327.22	316.92	322.50	330.65	342.74	353.74
	*P*_h_ (MPa)	19.42	19.79	19.14	19.90	19.32	19.56	19.71	19.86
	*W*_net_ (kW)	13.58	14.68	15.90	17.05	17.99	18.53	19.00	18.77
CO_2_/R601	*T*_3_ (°C)	341.40	333.92	326.84	326.39	326.58	318.00	344.03	353.74
	*P*_h_ (MPa)	19.42	19.36	19.02	19.46	19.42	19.63	19.32	19.86
	*W*_net_ (kW)	13.01	14.16	15.52	16.91	18.05	18.76	19.04	18.77

**Table 13:** Optimal cycle parameters of the split cycle at different CO_2_ mass fractions.

Fluids	Parameters	Mass Fraction of CO_2_							
		0.30	0.40	0.50	0.60	0.70	0.80	0.90	1.00
CO_2_/R290	*T*_3_ (°C)	344.68	338.32	330.55	361.19	324.35	323.84	319.98	321.57
	*P*_h_ (MPa)	12.01	12.39	14.48	17.31	12.84	15.70	15.98	17.16
	*SR*	0.67	0.73	0.69	0.61	0.71	0.70	0.68	0.62
	*W*_net_ (kW)	23.36	23.43	23.93	24.24	24.70	25.48	25.86	26.80
CO_2_/R600a	*T*_3_ (°C)	309.09	299.37	312.38	283.43	299.38	308.87	320.43	321.57
	*P*_h_ (MPa)	11.47	12.37	14.05	12.46	13.99	16.38	16.54	17.16
	*SR*	0.78	0.79	0.68	0.79	0.74	0.68	0.63	0.62
	*W*_net_ (kW)	21.70	22.28	22.91	24.91	26.01	26.63	26.95	26.80
CO_2_/R600	*T*_3_ (°C)	320.86	314.91	321.25	290.38	303.92	316.46	313.22	321.57
	*P*_h_ (MPa)	13.31	13.57	13.56	12.60	15.90	14.61	15.33	17.16
	*SR*	0.66	0.75	0.67	0.72	0.71	0.66	0.66	0.62
	*W*_net_ (kW)	19.25	20.41	21.84	24.03	24.79	26.17	27.43	26.80
CO_2_/R601a	*T*_3_ (°C)	330.30	336.88	320.03	285.92	306.95	286.34	321.87	321.57
	*P*_h_ (MPa)	12.51	14.13	14.83	14.13	13.66	14.07	14.78	17.16
	*SR*	0.74	0.66	0.74	0.74	0.69	0.76	0.66	0.62
	*W*_net_ (kW)	17.06	18.37	20.39	22.61	24.85	26.76	27.07	26.80
CO_2_/R601	*T*_3_ (°C)	334.36	327.62	341.49	310.13	300.58	312.56	324.49	321.57
	*P*_h_ (MPa)	13.20	13.71	14.89	13.38	14.19	13.56	17.10	17.16
	*SR*	0.63	0.76	0.68	0.73	0.73	0.64	0.63	0.62
	*W*_net_ (kW)	16.03	17.86	19.49	22.59	24.68	25.56	27.04	26.80

## Data Availability

Not applicable.

## Abbreviation

*c*_p_	Specific heat (kJ/(kg·K))
*I*	Exergy destruction rate (kW)
*W*	Power (kW)
*e*	The specific exergy of flow at any state point (kW/kg)
*SR*	Split ratio
*E*	Exergy (kW)
*Q*	Heat transfer rate (kW)
*h*	Specific enthalpy (kJ/kg)
*m*	Mass flow rate (kg/s)
*P*	Pressure (MPa)
*s*	Entropy (kJ/(kg·K))
*T*	Temperature (°C)
*η*	Efficiency (%)
Δ	Difference
ICE	Internal combustion engine
ORC	Organic Rankine cycle
GA	Genetic algorithm
PPTD	Pinch point temperature difference
GWP	Global warming potential
ODP	Ozone depletion potential
p	Pump
f	Mixture fluid
i	A state point of cycle
Re	Recuperator
g	Exhaust gas
in	Inlet temperature of exhaust gas
mid	Outlet temperature of Heater1
out	Outlet temperature of Heater2
con	Condenser
t	Turbine
L	Turbine out pressure
net	Net work
b	Normal boiling point
c	Critical state
h	Turbine inlet pressure
r	Recovery efficiency
th	Cycle efficiency
ex	Exergy
total	Total heat absorbed by the system

## Appendix A

**Table A1:** Thermodynamic properties of CO_2_/R290 at different state points of the recuperative cycle under design conditions.

State Point	*P*(MPa)	*T* (°C)	*s*(kJ/(kg·K))	*h*(kJ/kg)
1	3.27	30.00	1.40	294.91
2	13.00	40.98	1.42	317.50
2i	13.00	238.35	2.82	885.51
3	13.00	375.00	3.44	1238.91
4	3.27	316.68	3.51	1132.41
4i	3.27	59.45	2.25	564.41

**Table A2:** Thermodynamic properties of CO_2_/R290 at different state points of the split cycle under design conditions.

State Point	*P*(MPa)	*T* (°C)	*s*(kJ/kg·K)	*h*(kJ/kg)
1	3.27	30.00	1.40	294.91
2	13.00	40.98	1.42	317.50
2i	13.00	301.68	3.12	1047.50
3	13.00	375.00	3.44	1238.91
4	3.27	316.68	3.51	1132.41
4i	3.27	80.09	2.41	6214.14
5	13.00	40.98	1.42	3175.04
6	13.00	301.68	3.12	1047.50
7	13.00	301.68	3.12	1047.50
